# Long Timescale fMRI Neuronal Adaptation Effects in Human Amblyopic Cortex

**DOI:** 10.1371/journal.pone.0026562

**Published:** 2011-10-31

**Authors:** Xingfeng Li, Damien Coyle, Liam Maguire, Thomas M. McGinnity, Robert F. Hess

**Affiliations:** 1 Intelligent Systems Research Centre, University of Ulster, Derry, Northern Ireland, United Kingdom; 2 McGill Vision Research, Department of Ophthalmology, McGill University, Montreal, Quebec, Canada; University of Michigan, United States of America

## Abstract

An investigation of long timescale (5 minutes) fMRI neuronal adaptation effects, based on retinotopic mapping and spatial frequency stimuli, is presented in this paper. A hierarchical linear model was developed to quantify the adaptation effects in the visual cortex. The analysis of data involved studying the retinotopic mapping and spatial frequency adaptation effects in the amblyopic cortex. Our results suggest that, firstly, there are many cortical regions, including V1, where neuronal adaptation effects are reduced in the cortex in response to amblyopic eye stimulation. Secondly, our results show the regional contribution is different, and it seems to start from V1 and spread to the extracortex regions. Thirdly, our results show that there is greater adaptation to broadband retinotopic mapping as opposed to narrowband spatial frequency stimulation of the amblyopic eye, and we find significant correlation between fMRI response and the magnitude of the adaptation effect, suggesting that the reduced adaptation may be a consequence of the reduced response to different stimuli reported for amblyopic eyes.

## Introduction

Amblyopia, a condition in which there is a loss of visual function associated with either early misalignment of the visual axes (strabismus) or a refractive imbalance between the two eyes (anisometropia), has been extensively studied by brain imaging methods [1], [2], [3], [4], [5], [6], [7], [8], [9], [10]. Although brain imaging results have generally shown a decreased activation [2], [4], [5], [6], [7], [8], [9], [10], [11] and effective connectivity analysis of the functional magnetic imaging data (fMRI) [12] has also demonstrated deficits in the amblyopic cortex, little attention has been paid to the possible effects of differential neuronal adaptation between normal and amblyopic activation. Neuronal adaptation [13], [14], [15], [16] refers to a reduced neuronal response to repetitive stimulation, it is different form skill learning in that skill learning is measured as an improvement in the speed and/or accuracy of performance on a task with practice [17]. Neuronal adaptation has been observed in a number of studies including visual priming and working memory [18]. The basic idea of neural adaptation studies is that the neuronal system is plastic [14], and repeated stimulation with the same set of stimuli results in automation [15], [16] and decreased activity in task-related regions. With the advent of fMRI, it is possible to study the adaptation effect for cognitive tasks, such as those involved in visual perception, memory, and language [19]. Given the proven effectiveness of fMRI in brain mapping, it is now a standard tool with which to study the normal [20], [21] and amblyopic visual cortex [22]. Although orientation-specific fMRI adaptation [23] has been studied in the amblyopic cortex [22], little is know about the adaptation properties of the amblyopic cortex. There are specific findings from brain imaging that may be the direct consequence of a reduced adaptation response by the amblyopic cortex. There include 1. the unexpectedly subtle reduction of activation when driven by the amblyopic eye, 2. there is a general lack of correlation between the fMRI and psychophysical deficits to the same stimuli in amblyopia [24] that could potentially be explained if the amblyopic cortex exhibited less adaptation properties for the inputs from the amblyopic versus fellow fixing eyes in general and if this depended on stimulus spatial frequency in particular. Until we know more about the adaptation properties associated with the input from the amblyopic eye we will not be able to answer these questions.

The aims of this study are to use a commonly used stimuli presented in phase-encoded and random block fMRI experimental designs to investigate the adaptation properties of neuronal populations in the amblyopic cortex associated with the inputs from the amblyopic and fellow fixing eyes. We employ a wide range of stimuli including retinotopic mapping stimuli (wedge and polar angle stimuli) and stimuli of different spatial frequency (phase-encoded and random block designs) to address the question of adaptation effects in the amblyopic cortex because these stimuli have been used in past studies to delineate the fMRI deficit in amblyopia. Specifically, we addressed three important questions that are critical for interpreting fMRI data from human amblyopes. First, *is there reduced neuronal adaptation in the cortex driven by the amblyopic eye compared with that of the fellow fixing eye?* Previous fMRI studies compared the activation of fellow fixing and amblyopic eyes, assuming comparable adaptation effects. There is a suggestion from the previous study [22] that this assumption may not be correct. Second, *is the adaptation effect the same across all cortex regions?* Previous fMRI studies [1], [3] have compared amblyopic activation deficits in different visual cortex regions assuming comparable adaptation influences, we speculate that adaptation may show a regional dependence. Third, *is the adaptation effect for different stimuli simply a function of the strength of activation?* A previous magnetoencephalography (MEG) study [25] showed that adaptation strength is a function of response strength. fMRI studies have shown that spatio-temporal broadband retinotopic mapping [3] stimuli produce stronger response than narrowband spatial frequency stimuli [8]; we therefore hypothesize that fMRI neuronal adaptation will be greater for retinotopic mapping comparing with spatial frequency stimuli in the amblyopic cortex.

## Materials and Methods

### Subjects and MRI data collection

All studies were performed with the informed consent (consent statement was written) of the subjects and approved by the Montreal Neurological Institute Research Ethics Committee and followed the tenets of the Declaration of Helsinki. Three experimental designs were analyzed for the study. The first experiment was the standard retinotopic mapping experiment which involved eleven normal subjects (mean age is 33 years, the standard deviation is 5 years) and 11 amblyopic subjects (mean age is 34, the standard deviation is 15 years) (for more details regarding the imaging protocol and amblyopic subjects, see [3], [11]). Briefly, in this experiment, visual retinotopic mapping stimuli in a phase-encoded design [26], [27], [28] were used. Each visual retinotopic experiment (phase-encoded design, travelling square wave) consisted of four acquisition runs for each eye (two eccentricity runs, two polar angle runs, two clockwise order runs, and two counter-clockwise runs), with each of the 128 image volumes acquired at three second (TR = 3 s) intervals for the left and right eye of normal subjects. Runs were alternated between the eyes in each case while the subject was performing a task to maintain a constant level of attentional activation in the scanner. The second experiment involved a phase-encoded spatial frequency design (for details see [8]). Because we cannot get back all the subjects from retinotopic experiment to do the experiment, only five normal subjects (BM, BH, MM, PH, and RH) and 6 amblyopic subjects (EF, GN, HP, LM, GN, and XL) participated in this experiment. The phase-encoded design (the spatial frequency changed periodically either from high to low or from low to high) in which the spatial frequency of a sinusoidal checkerboard stimulus was gradually varied from 0.5 to 6 cpd over a 1 min period was used in the experiment. The temporal frequency of the checkerboard stimulus was 8 Hz. The cyclic change that occurred in spatial frequency from the lowest to the highest (and vice versa) over the 6-min run time. This involved a smooth and gradual change in the spatial frequency of the sinusoidal checkerboard evenly throughout the field. A central fixation point was provided. The attention of the subjects was controlled using a target detection task in which the appearance of a subset of checks (whose position and presentation was chosen randomly) of a higher local contrast/luminance had to be detected. Performance was not significantly different for amblyopic and normal eye stimulation (varied between 78 and 93%). The third experiment involved a random block design in which three conditions i.e., spatial frequency 0.25 cycle per degree (cpd) (low spatial frequency), spatial frequency 4 cpd (high spatial frequency), and mean luminance stimulus were presented randomly. The temporal frequency of the checkerboard stimulus was also 8 Hz. Each block lasted 15 s and there were eight blocks per run. Eight amblyopic subjects (DA, DV, GN, MB, LM, HP, OA, and XL) took part in this experiment (for details see experiment 1 of [8]). The high spatial frequency sinusoidal checkerboard stimulus of 4 cpd, a low spatial frequency of 0.5 cpd, and control condition (mean luminance) were presented randomly. The attention of the subjects was controlled using a target detection task as described above for the phase-encoded spatial frequency design. The same task was performed for test and control conditions. Performance varied between 80 and 97% for amblyopic and normal eye stimulation. In all three experiments, subjects viewed the stimuli monocularly and the eye that was not subjected to stimulation was occluded with a black patch that excluded all light from the eye. 128 volumes of fMRI data were collected for all experiments.

For the data pre-processing, dynamic motion correction for functional image time series for each run and for different runs were realigned at the same time by using the fmr_preprocess function (provided in the MINC software package: http://noodles.bic.mni.mcgill.ca/ServicesSoftware/HomePage) with default parameters of three-dimensional Gaussian low-pass filtering. The first eight scans of each functional run were discarded due to start-up magnetization transients in the data, so only 120 image volumes were used for each run ( for phase-encoded designs, 20 image volumes were used for each cycle, and 8 image volumes for each block were analyzed for the random block design).

## Results

### Within run analysis for retinotopic mapping stimuli

**Figure 1:**
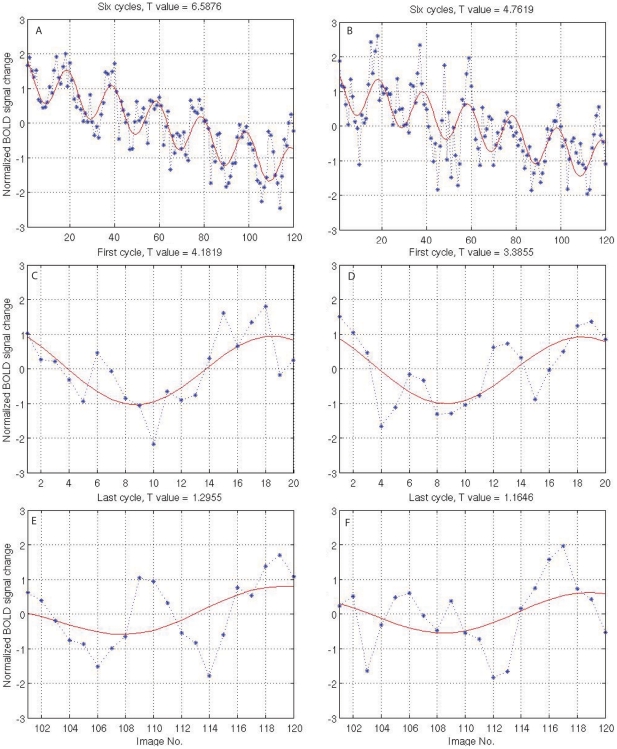
Two example fMRI response curves from an amblyopic subject (YC) at the same voxel position in V1 (right hemisphere). A, C, and E are the response driven by the fixing eye; B, D, and F are the responses driven by the fellow amblyopic eye. A is the overall fMRI response driven by the fixing eye. B is the overall response driven by the amblyopic eye. C is the local response of the first cycle of A. D is the local response of the first cycle of B. E is the local response of the last cycle of A. F is the local response of the last cycle of B.

Figure 1A shows one typical fMRI response with slow drift driven by the fixing eye of an amblyopic subject (YC) to the polar angle clockwise stimuli. Figure 1B shows one typical fMRI time series driven by the amblyopic eye of the subject (YC) to the same stimulus at the same voxel position in the cortex. Comparing Figure 1A with Figure 1B, it is clear that the fixing eye (Figure 1A, 1C, and 1E) has a better signal to noise ratio (SNR) than the amblyopic eye (Figure 1B, 1D, and 1F) quantified by the T values. In addition, the T values suggest that the first cycle response (Figure 1C and Figure 1D) is smaller than that of the overall time fMRI (whole time series in one fMRI run) response for the fixing and amblyopic eyes (Figure 1 A and B). The last cycle (6^th^ cycle) response is smaller than the first cycle response (compare Figure 1E:1F and 1D:1F) for the fixing and amblyopic eyes. Although the adaptation effects for fixing and amblyopic eye activation appear to be comparable, closer inspection of Figures 1C/1E vs 1D/1F suggests that adaptation has differential effects on the SNR of the response for the fixing and amblyopic eyes. For example, there is a larger difference between the activation of the fixing and amblyopic eyes (i.e. the amblyopic activation deficit) for the 1^st^ cycle of stimulation than there is for the last cycle of stimulation, suggesting less adaptation for the amblyopic compared with the fixing eye's input for this response. However, the effect is subtle.

**Figure 2 pone-0026562-g002:**
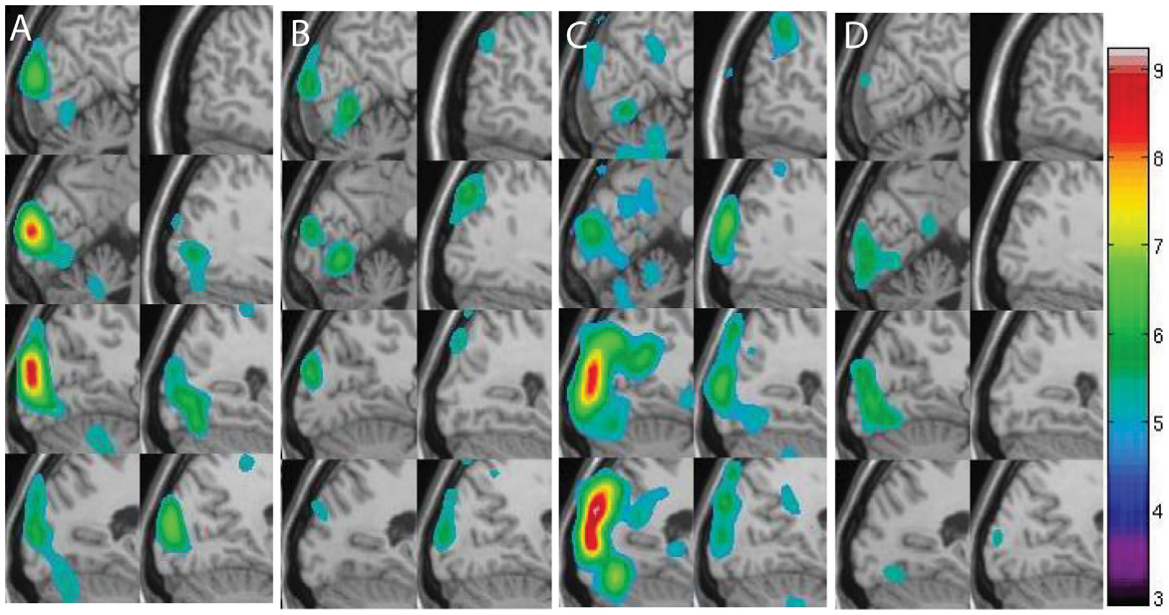
Activation of a typical functional run. A is the response driven by the amblyopic eye from the first cycle. B is the response driven by the amblyopic eye from the last cycle. C is the response driven by the fixing eye from the first cycle. D is the response driven by the fixing eye from the last cycle.

To quantify the magnitude of the adaptation effect, a hierarchical linear model for the fMRI data analysis is employed (see appendix S1 for detail). Because the t value is the ratio between an effect and standard deviation as shown in equation (5) or equation (17), using model coefficients ( equation (3)) alone may not be enough, thus, we adopted linear model to compare different groups/eyes for the analysis. Using a first level fMRI analysis (equations (1–5) in appendix S1) voxel by voxel, we obtain the activation map of one run in response to the polar angle clockwise stimulus from one amblyopic subject (YC) as shown in Figure 2. Figure 2 displays the adaptation effects projected on the structural MRI in Talairach space [29]. The left column of Figure 2A (Figure 2B and Figure 2C) is the sagittal section of the right side of the cortex including visual cortex (including calcarine sulcus). The right column of Figure 2A (Figure 2B and Figure 2C) is the right part of the cortex, including the visual cortex. The slice interval is 10 mm in Figure 2 and the observed adaptation effects are extensive in the visual cortex. Color regions in Figure 2A show the activation for the first stimulus cycle and Figure 2B shows the same run for the last stimulus cycle of subject YC. Figure 2C shows the same amblyopic subject driven by the fixing eye in response to the same stimulus. Figure 2D shows the corresponding last cycle response. In Figure 2, the false discovery rate (FDR) [30] method was employed to correct the threshold. The colour regions show that the activation is significant (P<0.05). It is clear that the first cycle responses (Figure 2A and Figure 2C) are stronger than the last cycle responses (Figure 2B and Figure 2D), suggesting an adaptation effect in the fMRI response. From Figure 2 we can see that the results show a larger adaptation by the fixing eye in response to this stimulus. In addition, the results indicating the stronger the response, the bigger is the adaptation effect.

### Regional adaptation effect analysis from retinotopic mapping stimuli

**Figure 3 pone-0026562-g003:**
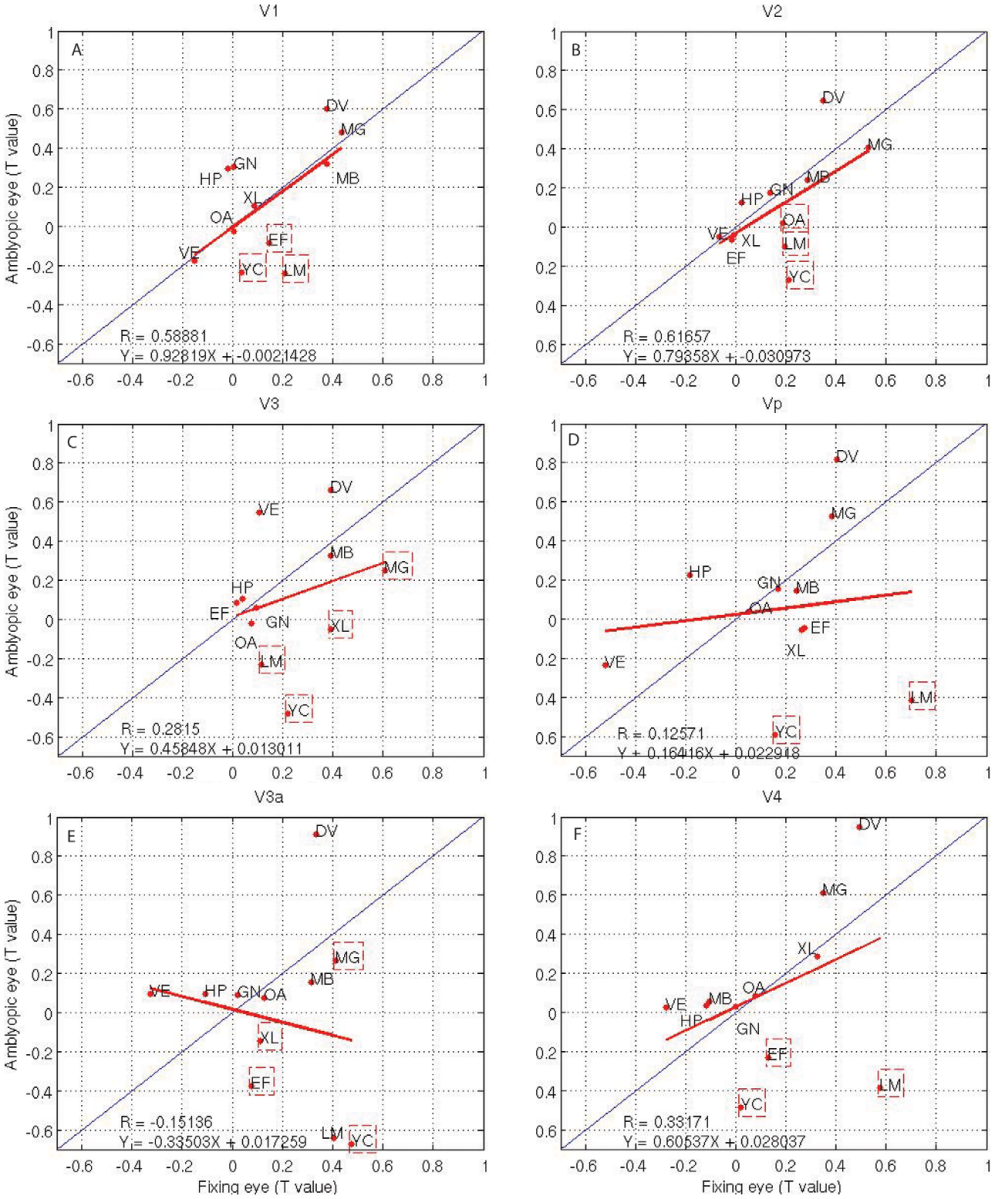
Amblyopic brain adaptation (T statistic) for fixing versus amblyopic eye in amblyopic subjects; Data from subjects whose initials are within dashed squares are significant (T>1.960; P<0.05, two tailed t-test). Thin line: represents equal activation; bold solid line: the robust fit to the amblyopic data as a whole. R is the correlation coefficient. Regression equation is 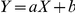, where 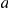 is slope.

**Figure 4:**
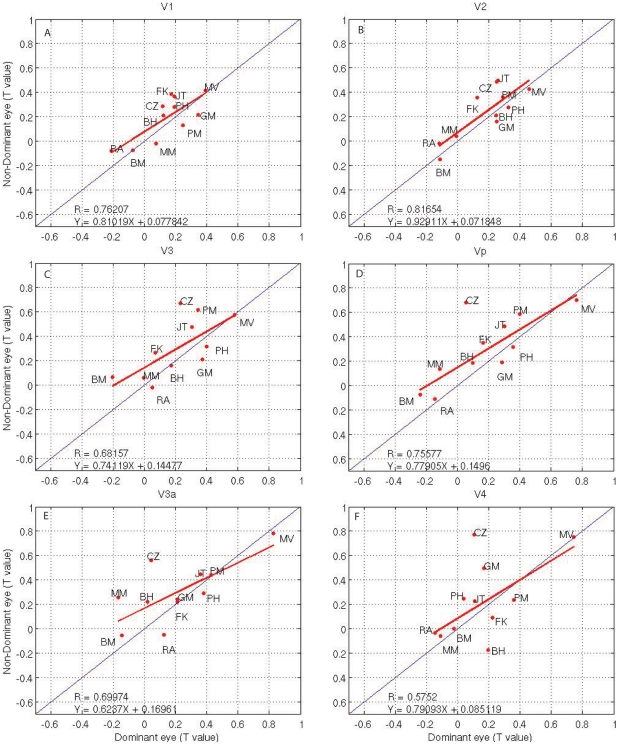
Brain adaptation activation (t statistic) from dominant versus nondominant eye stimulation in normal subjects; the slope of the thin line is 1; the slope of the bold solid line is estimated by using robust regression method to the data. Regression equation is 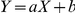, where 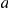 is slope.

To investigate the regional adaptation effects in the amblyopic cortex, we plot comparisons of adaptation in different cortical areas for amblyopes (Figure 3) and normal subjects (Figure 4) in a way in which the contribution from individual subjects can be identified. In Figure 3, the t statistic for the adaptation of the amblyopic and fixing eyes of amblyopic subjects is plotted against each other. In each of these and subsequent figures, the data for each subject are identified by his or her initials. The bold solid line is the best-fitting line to the population as a whole and, in all brain areas investigated. R represents the correlation coefficient between responses from the two eyes. A similar comparison is shown in Figure 4 for the normal control population, with the data for each control indicated by initials. The best-fitting line to the amblyopic population (bold line) can be compared with the unity prediction (thin line) found for normal subjects (Fig. 4). The slope values and its regression equation are shown in Figure 3 (bottom left).

Because the large inter-subject variability evident in Figures 3 and 4 for the amblyopic and normal populations limits the sensitivity of the group comparisons (i.e., either in terms of the slopes in Figure 3), we assessed the significance (volume of interest [VOI] paired t-test; fixing versus amblyopic eye, P<0.05) of the reductions in cortical adaptation effect for each amblyopic subject separately using the fellow fixing eye as reference. The advantage of such a comparison is that each subject can act as his or her own control, with a subsequent reduction in variability. The disadvantage is that the fellow fixing eye's adaptation may be slightly reduced below that of the dominant eye of a normal observer and as a consequence, any adaptation difference found between the amblyopic and fixing eye would underestimate the extent of the amblyopic dysfunction. In Figure 3, for each visual area, we have indicated subjects (by enclosing initials in a dashed box) whose reduced cortical adaptation, when driven by their amblyopic eye, was statistically significant. Because it appears that several visual cortical areas that we mapped have reduced adaptation if driven by the amblyopic eye, we wondered to what extent the extrastriate loss correlates with the striate loss. The reduced V1 adaptation, as quantified by the t statistic difference between adaptation of fellow fixing and amblyopic eyes, is calculated against the reduced adaptation in other visual cortical areas (i.e., V2, V3, Vp, V3a, and V4, all significant P<0.05), suggesting that the striate and extrastriate adaptation losses are significantly correlated in all mapped areas. Comparing plot 3A to the other plots in Figure 3, we found a greater adaptation deficit in extra-cortex regions than in V1 in terms of regression slope. In addition, comparing Figure 3 with Figure 4, we can see that the correlation coefficients for the normal controls are larger than the corresponding amblyopic subjects in all cortical areas. Although the slope of the regression line in amblyopic subjects in V1 is slightly larger than corresponding control groups, the other cortex regions are smaller than healthy controls, indicting adaptation deficits in the extra-cortex areas of amblyopic subjects.

### Between subject analysis for retinotopic mapping stimuli

**Figure 5:**
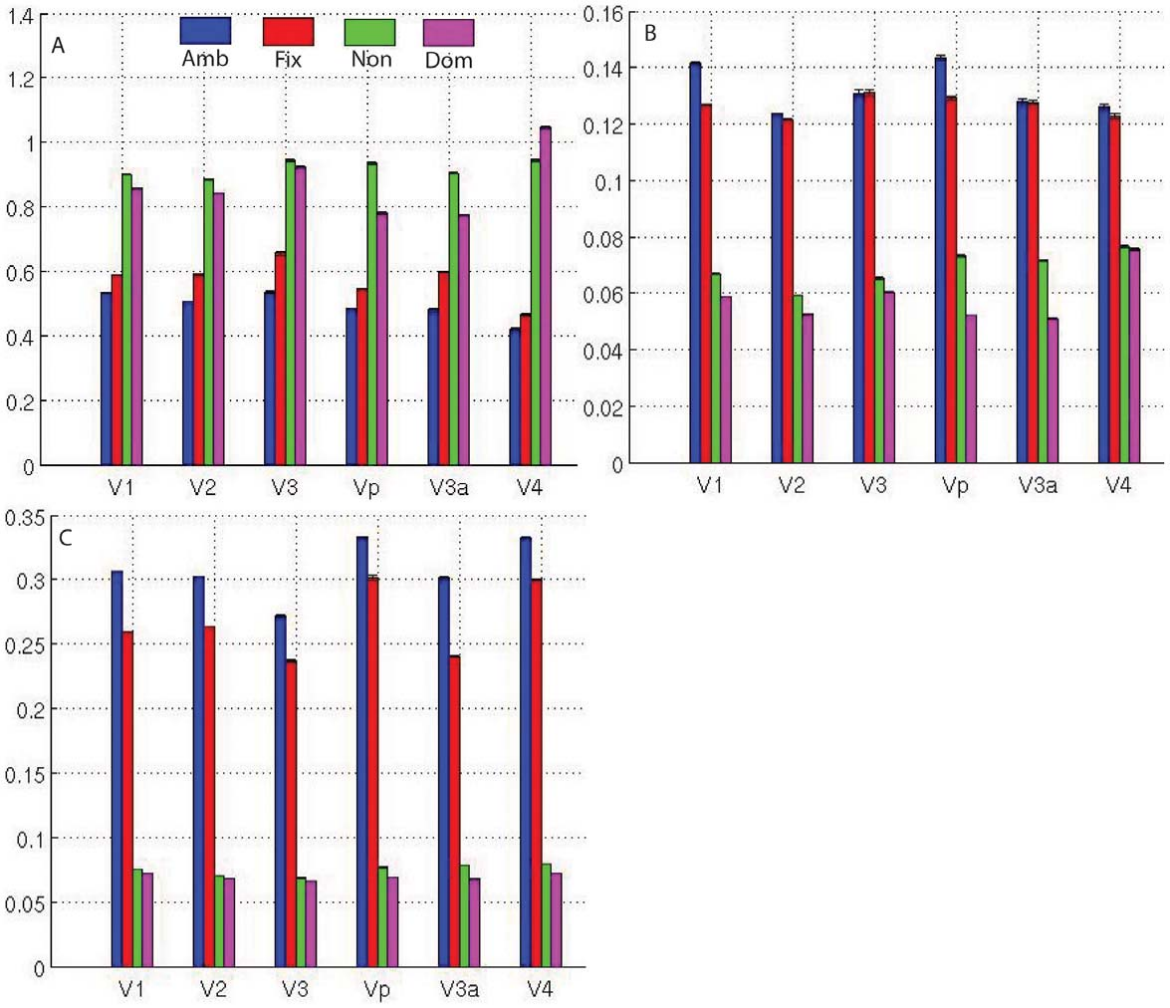
Adaptation effect and its statistics analysis for the retinotopic mapping experiment. A, B, and C is the T value, effect value, and variance values respectively.

To study the adaptation effect, we can compare the first cycle response with the last cycle response. This is achieved by a second level analysis in hierarchical linear model as described in the appendix S1. In the second level analysis, different subjects' results were combined with the design matrix as in equation (18) within the mixed effect model (equation (6)), and these results are given in Figure 5. Because it is a between subject design, the visual cortex template obtained from automatic volumetric segmentation [31] was used to define each region for this comparison. Blue bars in Figure 5 represents the amblyopic eyes' adaptation effect (Figure 5A, and its effect map Figure 5B and variance map Figure 5C) across all the amblyopic subjects and red bars show comparable results for the fixing eye. The group adaptation effect of the nondominant eye of normal subjects is shown in green, and magenta bars display the group adaptation effects of the dominant eye of the same normal group. From a comparison between the blue (amblyopic eye) and red bars (fixing eye), it is obvious that the fixing eye has a slightly stronger adaption effect (in terms of T value in Figure 5A) than the amblyopic eye but the striking result is that normal subjects have stronger adaptation effects than either eye in the amblyopic group (Figure 5A), suggesting there is a reduction of adaptation in the cortex of amblyopes when driven by either the fixing or the amblyopic eye. From the statistical comparison, a two-sample test was adopted to test if the T value was larger than zero which would indicate that there was an adaptation effect within each cortex region. We found that the regional adaptation differences between the eyes (blue and red bars in Figure 5A) of amblyopes (differences between the first cycle and last cycle response) was significant (P<0.05, T>1.96) in all visual cortex except V4, indicating significant reduction of adaptation in these regions in the amblyopes' cortex when driven by the amblyopic eye regardless of which eye was activated.

From Figure 5, we can see that the cortical response in term of T statistics, due to amblyopic eye stimulation is large yet variable. We display the t map (Figure 5A), the corresponding effect map (coefficient map, Figure 5B) and the variance map (Figure 5C). It is evident that the effect map (which is the coefficient, see equation (3)) of amblyopic eye is larger than the fixing eye (Figure 5B). However, the corresponding standard deviation map (Figure 5C) is much larger than the fixing eye. As a result the t value (Figure 5A), which is the ratio between an effect and standard deviation, as shown in equation (5) or equation (17), is smaller for the amblyopic eye. This result illustrates the use of response coefficient alone for the statistics analysis can cause confusion.

### Adaptation effect differences for retinotopic mapping stimuli, phase-encoded spatial frequency stimuli, and random block spatial frequency stimuli

**Figure 6:**
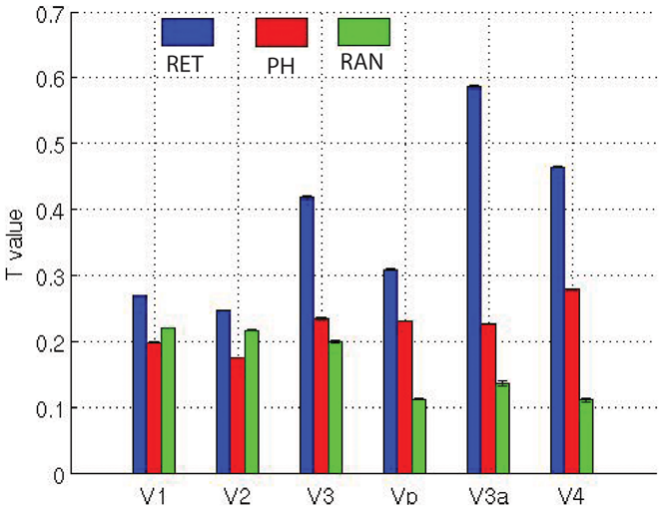
Differences in the adaptation effects between controls and amblyopic eyes from retinotopic mapping, phase-encoded spatial frequency, and random spatial frequency design stimuli. RET, Differential adaptation effects between normal compared with amblyopic subjects in response to retinotopic mapping stimuli. PE, for phase-encoded spatial frequency stimuli response. RAN, a similar comparison for the 0.25 cpd and 4 cpd spatial frequency stimulus presented in a random block design.

To study the adaptation effect from different stimuli, we have compared the amblyopic subjects with normal controls within the mixed models (as given in equation (6) of appendix S1) by defining the design matrix in equation (7) and equation (19) of appendix S1 for the group comparison. The results show that the control subjects exhibit stronger adaptation than that of the amblyopic subjects as indicated in Figure 6. All visual cortical regions show larger adaptation for controls than for amblyopes.

To study the spatial frequency adaptation properties, we analyzed the data in the same way as retinotopic experiments but for the phase-encoded spatial frequency experiment. Eleven subjects (five normal subjects (BM, BH, MM, PH, and RH) and 6 amblyopic subjects (EF, GN, HP, LM, GN, and XL) participated in this experiment. We compared the 1^st^ and last cycle responses for each eye of control and amblyopic subject. In the first level analysis, the first cycle and last cycle responses were compared in the same way as the retinotopic mapping stimuli. In the second level analysis, different spatial frequency runs were combined based on equation (18) of the appendix S1 for each eye. Then, equation (19) in the appendix S1 was used to compare normal subjects with the amblyopic subjects for the third level data analysis. The final results for spatial frequency adaptation effects in the early visual cortex are displayed by the red bars in Figure 6. It shows that the adaptation differs between normal and amblyopic subjects in the visual cortex (red bar in Figure 6).

To make a similar comparison of response for spatial frequency, we combined responses to low and high spatial frequency stimuli presented in a random block design as equation (18) in appendix S1 for the second level analysis. Because only 8 amblyopic subjects participated in this experiment, we only performed the first and second level analysis for this set of data, i.e., a within group comparison. In the first level of the data analysis, we combined different runs in the same way as described above for retinotopic and phase-encoded spatial frequency experimental designs. In the second level analysis, we combined the 0.25 cpd (high spatial frequency) and 4 cpd (low spatial frequency) frequency responses within the design matrix as given in equation (19) of the appendix S1. The regional adaptation effect is represented by the green bars in Figure 6.

From the results shown in Figure 6, it is obvious that broadband retinotopic mapping stimuli produced stronger adaptation effects compared with narrowband spatial frequency stimuli in all visual cortex areas. However, this does not reach significance in area of V2. The results show that there is a difference in the adaptation effects for control and amblyopic eyes for spatial frequency stimuli presented in a phase-encoded design that are significantly (P<0.05, T>1.96) larger than that spatial frequency stimuli presented in a random block design in areas of Vp, V3a, and V4.

### fMRI response correlated with adaptation effect from retinotopic mapping stimuli

To investigate the relationship between fMRI responses and adaptation effect, we calculate the correlation coefficients between fMRI response and adaptation effect for each eye. The results are given in Table 1. We found that most cortical regions exhibit a significant correlation between the adaptation for both amblyopic and controls and the strength of fMRI activation (Table 1). These results suggest that the reduced adaptation effects in the amblyopic subjects may be a consequence of reduced fMRI activation. In addition, from Figure 5, we found that normal subjects have a stronger fMRI response than their amblyopic counterparts; this may be why normal eyes exhibit a stronger adaptation effect in Figure 5. Furthermore, in Figure 6, we found the retinotopic mapping stimuli produce stronger adaptation effects, which may be simply due to the fact that retinotopic mapping evoke stronger activation than the spatial frequency stimuli.

**Table 1:** Correlation analysis for adaptation effect with fMRI responses.

	V1	V2	V3	Vp	V3a	V4
Amblyopic	**0.5411** *	**0.4606** *	0.3566	**0.6294** *	**0.5123** *	**0.6128** *
Fixing	**0.4441** *	**0.5306** *	**0.4852** *	**0.7270** *	**0.5837** *	**0.6557** *
Dominant	**0.7305** *	**0.6125** *	**0.8022** *	**0.7390** *	**0.5516** *	**0.6193** *
Non-Dominant	0.2341	0.3011	**0.6398** *	**0.5619** *	**0.4788** *	**0.6058** *

*P<0.05 (R>0.423, df = 20, Two-Tailed Test).

## Discussion

We are mainly interested in the fMRI response to the first cycle and the later cycle of the stimuli, as the response differences between first and later cycle of the stimulus reflects fMRI adaptation. From physiology studies [32], we know that the adaptation effect is stronger during prolonged stimulation. Based on this observation, we compared the first cycle and the last cycle responses to quantify adaptation effect in the amblyopic cortex and our results can be summarize as follows.

First, our results demonstrate that there are subtle adaptation differences between control and amblyopic subjects in different areas of the visual cortex and also between amblyopic and fellow fixing eyes in different areas of the visual cortex. Normal control subjects and the fellow fixing eyes of amblyopes exhibited greater adaptation. It is well-known that adaptation strength is correlated to the fMRI response strength in the visual cortex (e.g., Figure 1–4), so this may follow as a consequence of the small fMRI response differences (activation differences) for this stimulation. Our study of the adaptation effect is based on the use of random block and phase-encoded designs, which operate over a longer timescale than event-related (ER) designs [21], [33]. Furthermore, it is also clear that the adaptation effect in different visual cortical regions is different in response to the different stimuli (Figure 6). Because the viewing duration is same for both retinotopic stimuli and phase-encoded spatial frequency design, retinotopic stimuli produce slightly stronger adaptation effects than do spatial frequency stimuli. This could also be due to the retinotopic stimuli having much stronger fMRI responses due to their spatio-temporal broadband structure than the spatial frequency stimuli in this study, and the adaptation effect being correlated with the fMRI response.

Second, the timescale effect for the fMRI adaptation was investigated based on spatial frequency stimuli presented in a random block design. We found the timescale for the adaptation effect has a greater effect on the amblyopic eye than for the fixing eye. There is a suggestion that adaptation is greater in the amblyopic eye at the longer timescale for spatial frequency stimuli but it falls short of reaching significance. Furthermore, we found adaptation effects in the fixing eye are consistent compared with the amblyopic eye for the 5 minutes timescale (and at least 15 seconds for the random block design). This suggests that the timescale for the adaptation effect is an important factor for studying these phenomena. One possible explanation for larger response variability is that the amblyopic eye lacks temporal stability as suggested in a psychophysical study [34].

Previous amblyopia studies [1], [2], [3] found reduced fMRI responses in the visual cortex when driven through the amblyopic eye but these studies have not addressed the possible role of adaptation in amblyopic subjects. Recently, an experiment [22] was designed to combine the paradigms of fMRI adaptation and interocular transfer of adaptation as a consequence of *dichoptic* visual stimulation. They found amblyopic subjects showed consistent *monoptic*, but no *dichoptic* adaptation in the V1 area and extrastriate cortical regions. This is consistent with our results of V1 and extrastriate cortex adaptation in response to the monocular stimuli. In the data analysis, Jurcoane et al [22] compared the fMRI response peaks to quantify the adaptation effect, as shown in Figure 1 (absolute magnitude of fMRI response in Figure 1F is larger than Figure 1E and 1D) although this method could lead to estimation bias. In contrast, our method includes both peak/magnitude and shape information in the data analysis, and therefore, provides more statistical power.

Finally, because the stimuli used in our study include motion, orientation, and spatial frequency, we assume their adaptation effects act independently.

### Advantages and limitations of the method

One of the strengths of this study is that we employed a hierarchical linear model to analyze individual fMRI response changes for studying neuronal adaptation. As a result, the diverse repeated measure data patterns such as phase-encoded design fMRI data can be combined into a single analysis (appendix S1). The fMRI neuronal response is regarded as one longitudinal dataset [35], therefore a hierarchical model [36] can be employed to detect time-dependent changes, as these subjects are measured repeatedly across time. Focusing on characterizing the fMRI response decline/growth across time, we can study the adaptation effect. In addition, our method is based on a mixed effect model, therefore, both random and fixed effects can be taken into account, and the restricted maximum likelihood (REML) method is applied to estimate the parameters. Secondly, the current methods proposed to study fMRI adaptation require a special experimental design [22], [37], [38], [39], which limits its general application. In contrast, our method does not require a special experimental design, thus, it can be used for adaptation and block design experiments in general, as long as there are repeated stimuli presented. Another advantage of this study is that we employed a wider variety of stimuli, including retinotopic mapping stimuli (wedge stimulus, polar angle stimulus), phased-encoded spatial frequency stimuli, and random block low/high spatial frequency stimuli. Therefore, our results can be generalized across stimuli and experimental designs.

We applied the mixed effect model in the analysis, it has the advantage of taking into account fixed effect (within subject variance) and (individual) random effect (cross-subject variance) at the same time, e.g., equation (8). At the subject level, the variance includes both random and fixed effect; therefore, we consider the propagation of T value from the first level to the second level, while it is difficult to study the response propagation in term of coefficient. For instance, for the T statistics propagation, if the within subject variance is large, the 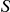 in equation (8) will be large, as a result, the T value will be small. Our result (Figure 5) suggests that analysis of the coefficient of the GLM is not enough to explore the fMRI adaptation effect, more complex statistical methods such as variance analysis should be applied to study the adaptation effect from the longitudinal dataset.

The major limitation of this study is that we cannot address the adaptation effect within the timescale of a second. This is because each task/condition/block is 1 minute for the phase-encoded design and at least 15 sec for the random block design. The nature of these experimental designs make it is difficult to study the short time (within second) adaptation effects in the amblyopic cortex. Our stimuli are longer than that of previous studies because we need 1 minute to present one cycle for the phase-encoded designs and 15 s for the random block design. It should be noted that when the first and last cycle/block of the response are compared, there are 5 minutes between the first stimulus and the last stimulus in our retinotopic mapping and spatial frequency stimuli. Therefore these results can only be considered as long timescale adaptation [20]. Moreover, because only 120 image volumes with temporal resolution of 3 s (TR = 3) were used for adaptation study, the fMRI time series may be too short for longer time scale adaptation effects.

In conclusion, the investigation was aimed at addressing the adaptation effect in amblyopic subjects and the data provides the following answers to the three posited questions: (1) *is there reduced neuronal adaptation in the cortex driven by the amblyopic eye compared with that of the fellow fixing eye?* The answer to this question is yes. We found a reduced fMRI adaptation in amblyopic cortex in response to the retinotopic stimuli. The amblyopic eye activation exhibits less adaptation than that of the fellow fixing eye but more importantly, the amblyopic cortex in general exhibits less adaptation than the cortex of normal observers. (2) *is the adaptation effect the same across all cortex regions?* Our results show different deficits in different visual cortical regions, and their adaptation regional contribution is different. We also found that more adaptation deficit was presented for retinotopic mapping stimuli in extra-cortex regions than in V1, suggesting different adaptation effects in different areas of the amblyopic cortex. (3) *is the adaptation effect for different stimuli simply a function of the strength of activation?* Our results show that the adaptation effects correlates with fMRI responses, indicating that reduced adaptation may be a consequence of reduced initial activation.

### Future work

Adaptation analysis provides useful information to improve our understanding of neuron properties in the amblyopic cortex. To extend this work, we propose to compare the current data for phase-encoded and random block designs with that from ER designs. In this way, the adaptation effects at short timescales (in second) can be investigated and compared with longer adaptation effects. Secondly, it would be interesting to compare adaptation effects at different stimulus contrasts in the amblyopic cortex, as the contrast loss in the amblyopic cortex is selective for higher contrasts [7]. Thirdly, it would be worthwhile investigating the relationship between adaptation effects and effective connectivity anomalies in the amblyopic cortex to better understand the role adaptation plays in the signal transmission between different cortical areas. Finally, the method for quantification of neuron adaptation effects can be applied to study other pathologies such as post stroke plasticity and Alzheimer's disease.

## Supporting Information

Appendix S1 **Hierarchical linear model to quantify fMRI neuronal adaptation.**
(DOC)
