# Supplementary material for: Long Timescale fMRI Neuronal Adaptation Effects in Human Amblyopic Cortex
Source: PLoS One. 2011 Oct 31;6(10):e26562. doi: 10.1371/journal.pone.0026562 (PMC3204980; doi:10.1371/journal.pone.0026562)
Supplement: Appendix S1 — Hierarchical linear model to quantify fMRI neuronal adaptation. (DOC) [file pone.0026562.s001.doc]

**Appendix S1: hierarchical linear model to quantify fMRI neuronal adaptation**

***First level (within subject) analysis***

For the fMRI time series from the phase-encoded and standard/random block designs, we can use the first cycle (block) of the response for comparison with the later/last cycles (blocks) of the response, to address the adaptation effect. In this study, for the reason of simplicity, we compare the first cycle with the last cycle to estimate the adaptation effect in the phase-encoded experiments. For the first and last cycles of each run, we can apply the general linear model (GLM) to quantify the response. Considering a design matrix including hemodynamic model $u_{i}$ and linear drifts in the model, we have [1]:

(1)

where$\beta_{1}$ is the interception, $x_{i,1}$is the linear slope drift, $i$ is the fMRI image frame, $Y_{i}$is the fMRI response; $\beta_{1}$ and $\beta_{2}$ are the associated coefficients of interception and slope drift, respectively. $u_{i}$ is the hemodynamic response model which can be estimated by the Fourier fundamental frequency of the fMRI response in the phase-encoded design. For block design experiment, $u_{i}$can be approximated by a block function convolved with a Gaussian function [2].

Suppose the error term $\varepsilon_{i}$ is autocorrelated [1,3], i.e. , where independently. Then we have:

(2)

where , ; , ; ; , , $i=2,\cdots,n$, where $n$is the number of fMRI image frames within each cycle for adaption comparison. The least squares estimation of $\beta$ is: , where is the pseudoinverse of

, and .

The vector of the residuals $R=(r_{1},\cdots,r_{n})'$ is , , where is the degree of freedom (df) ; . To inference an effect:

(3)

where $c$ is the contrast matrix $c=[1,0,0]$ for one input fMRI activation detection experiment, and 1 corresponds to the hemodynamic model (or brain system input) $u_{1}$.

(4)

where , , and the T statistic is:

(5)

***Second level (between runs) analysis***

For the second level analysis, the general linear mixed model (GLMM) [4,5,6] was adopted, i.e.:

(6)

where $E=(E_{1},\cdots,E_{n})$,$S=(S_{1},\cdots,S_{n})$ and $\eta$is normally distributed with zero mean and variance independently for $j=1,\cdots,n$. We will use the restricted maximum likelihood [7,8] (REML) algorithm to estimate .

In the adaptation analysis, since we want to compare the first cycle/block effect with the later/last cycle/block effect in the fMRI response, we define a design matrix $Z$as:

(7)

where ,,, and ; $n_{1}$ and $n_{2}$is the number of the first and last cycle respectively. To estimate covariance components, the expectation maximization (EM) algorithm [8,9] and its modified version [1] is used and given as follows:

Let **S =** and **I** be the $n\times n$identity matrix, $n=n_{1}+n_{2}$; Then the variance matrix of the effects vector **E** =is, (from (6) ):

**S**+ **I** (8)

In the numerical implementation of the algorithm, we subtract min($S_{j}$) from **S**, and add it back after we have estimated the values. In this way, the error in the numerical analysis can be reduced. To estimate the random effect, we define the weighted residual matrix:

**R**=**Z**(**ZZ**)**Z** (9)

where **Z**=$(z_{1},\cdots,z_{n})$. We start with an initial value of **ER_I_E/**$v$ assuming that the fixed effects variances are zero. The updated estimation is:

= (($p$+ tr(**SR**)) + **ERE**)/ (10)

where $p=\text{rank}(Z)$. Replace with in (8) and iterate (8-10) to convergence. In this work, 10 iterations appear to be enough [1]. In addition, because of numerical noise, we smooth the random effect as follows:

(11)

where $v_{j}$is the degree of freedom of $S_{j}$and $v_{f}=\sum_{j} v_{j}$.

(12)

We use the Gaussian function with full width at half maximum (FWHM) = 10 mm within this study for the smoothing operation in equation (12). Then, replacingby in equation (8), the estimation of $\gamma$is:

(**Z Z**) **Z E**  (13)

Its estimated variance matrix is:

(**Z Z**) (14)

In cases where the variances of $E$are not homogeneous across the second level analysis( i.e., the same physician did not collect the data), the above equation should be replaced by [6,10]:

(**Z Z**) **Z**(**EZ**)(**EZ**)**Z**(**Z Z**) (15)

Finally, the effect defined by a contrast matrix **b** in $\gamma$ can be estimated by $E$= **b** with standard deviation:

= (16)

and the T statistic is:

(17)

with a nominal degree of freedom () used to detect the effect.

***Third level (between subjects) analysis***

Besides comparing the different effects, if we are interested in the combination of different runs/subjects, we can set the contrast matrix to be: **b** = 1 and the new design matrix as:

(18)

where $n_{s}$is the total number of subjects. Then we perform the calculation from equation (8) to equation (10). If we are interested in different group comparison, we can set the design matrix as in equation (7) and set the corresponding contrast matrix as **b** = [1 -1]. For the group comparison, e.g. control subjects compared with amblyopic subjects in this study, the design matrix can be set as:

(19)

where $I_{1}=I_{2}$is a **1** element matrix with a size of $n_{1}\times1$, $O_{1}=O_{2}$is a **0** elements matrix with size of $n_{1}\times1$; $I_{3}=I_{4}$is a **1** elements matrix with a size of $n_{3}\times1$, $O_{3}=O_{4}$is **0** elements matrix with a size of $n_{4}\times1$;$n_{1}$ and $n_{3}$is the total number of normal and amblyopic subjects respectively. The contrast matrix can be set to **b** = [1 1 -1 -1] for comparing control subjects with amblyopic subjects accordingly.

**References**

1. Worsley K, Liao CH, Aston J, Petre V, Duncan GH, et al. (2002) A general statistical analysis for fMRI data. NeuroImage 15: 1-15.

2. Smith A, Singh KD, Williams AL, Greenlee MW (2001) Estimating receptive field size from fMRI data in human striate and extra-striate cortex. Cereb Cortex 11: 1182-1190.

3. Bullmore E, Brammer MJ, Williams SCR, Rabe-Hesketh S, Janot N, et al. (1996) Statistical methods of estimation and inference for functional MR images analysis. Magn Reson Med 35: 261-277.

4. Breslow NE, Clayton DG (1993) Approximate inference in generalized linear mixed models. Journal of the American Statistical Association 88: 9-25.

5. Bryk AS, Raudenbush SW (1992) Hierarchical linear models: applications and data analysis methods. New Delhi 110 048 India: SAGE Publications India Pvt. Ltd.

6. Sullivan LM, Dukes KA, Losina E (1999) Totorial in biostatistics: An introduction to hierarachical linear modelling. Statist Med 18: 855-888.

7. Dempster AP, Laird NM, Rubin DB (1977) Maximum likelihood from incomplete data via the EM algorithm. Journal of the Royal Statistical Society, Ser B, 39: 1-38.

8. Laird N, Lange N, Stram D (1987) Maximum likelihood computations with repeated measures: Application of the EM algorithm. Journal of the American Statistical Association 82: 97-105.

9. Laird NM, Ware JH (1982) Random-effects models for longitudinal data. Biometrics: 963-974.

10. Liang KY, Zeger SL (1986) Longitudinal data analysis using generalized linear models. Biometrika 73: 13-22.
